# Rapid and Effective Generation of Nanobody Based CARs using PCR and Gibson Assembly

**DOI:** 10.3390/ijms21030883

**Published:** 2020-01-30

**Authors:** Stijn De Munter, Alexander Van Parys, Layla Bral, Joline Ingels, Glenn Goetgeluk, Sarah Bonte, Melissa Pille, Lore Billiet, Karin Weening, Annick Verhee, Jose Van der Heyden, Tom Taghon, Georges Leclercq, Tessa Kerre, Jan Tavernier, Bart Vandekerckhove

**Affiliations:** 1 Department of Diagnostic Sciences, Ghent University, 9000 Ghent, Belgium; 2 Cytokine Receptor Laboratory, Flanders Institute of Biotechnology, VIB-UGent Center for Medical Biotechnology, Faculty of Medicine and Health Sciences, Ghent University, 9000 Ghent, Belgium; 3 Department of Internal Medicine and Pediatrics, Ghent University, 9000 Ghent, Belgium

**Keywords:** nanobody, VHH, chimeric antigen receptor, CAR T cell, CD33, CD20, PCR, Gibson Assembly, nanoCAR

## Abstract

Recent approval of chimeric antigen receptor (CAR) T cell therapy by the European Medicines Agency (EMA)/Federal and Drug Administration (FDA) and the remarkable results of CAR T clinical trials illustrate the curative potential of this therapy. While CARs against a multitude of different antigens are being developed and tested (pre)clinically, there is still a need for optimization. The use of single-chain variable fragments (scFvs) as targeting moieties hampers the quick generation of functional CARs and could potentially limit the efficacy. Instead, nanobodies may largely circumvent these difficulties. We used an available nanobody library generated after immunization of llamas against Cluster of Differentiation (CD) 20 through DNA vaccination or against the ectodomain of CD33 using soluble protein. The nanobody specific sequences were amplified by PCR and cloned by Gibson Assembly into a retroviral vector containing two different second-generation CAR constructs. After transduction in T cells, we observed high cell membrane nanoCAR expression in all cases. Following stimulation of nanoCAR-expressing T cells with antigen-positive cell lines, robust T cell activation, cytokine production and tumor cell lysis both in vitro and in vivo was observed. The use of nanobody technology in combination with PCR and Gibson Assembly allows for the rapid and effective generation of compact CARs.

## 1. Introduction

CARs are synthetic chimeric receptors consisting of an antibody based extracellular part to recognize specific antigens expressed on the surface of tumor cells and an intracellular part containing (co)stimulatory signals derived from CD3ζ, CD28 and/or 4_1BB [1]. Adoptive immunotherapy with chimeric antigen receptor (CAR)-modified T cells targeting CD19 have shown unprecedented response rates not only in relapsed/refractory acute lymphoblastic leukemia but also in diffuse large B cell lymphoma, chronic lymphocytic leukemia and multiple myeloma. These response rates were accompanied by durable complete remissions even after a single infusion of CAR T cells [2,3,4,5,6,7,8,9,10,11]. Current CAR T cells not only target CD19, but also other B cell-related antigens and antigens associated with non-B cell malignancies [12]. CAR T cells are administered as a “living drug” treatment. They undergo typical expansion, contraction and re-expansion in vivo upon exposure to the antigen and are able to persist for several years as memory cells [13]. 

CARs that obtained market authorization and those that are clinically tested rely on the generation of a monoclonal antibody, the sequencing of the variable heavy (V_H_) and variable light (V_L_) regions and the generation of a single-chain variable fragment (scFv) for target recognition [12]. While scFvs are typically capable of binding their antigens with high affinity, there are some drawbacks. First off all, a scFv can lose its binding affinity after conversion from an immunoglobulin (IgG) [14,15]. Secondly, the generation of a functional scFv can be cumbersome due to difficulties in finding the most optimal linker and V-region orientation [16]. Furthermore, mouse-based scFvs can induce an anti-mouse immune response. This immunogenicity can lead to serious adverse events, loss of CAR T cells and thus failure of CAR therapy [17,18]. Next, Long et al. have shown that interactions within the scFv framework of the CAR can induce CAR clustering on the cell membrane. This results in tonic CAR CD3ζ phosphorylation, which in turn induces early exhaustion of CAR T cells that limit antitumor efficacy [19]. This is in line with others who have reported on the strong tendency of scFvs to self-aggregate [20,21]. Finally, clinical trials have shown that immune escape is one of the leading causes of relapse after CAR T cell infusion [22]. Targeting two or more antigens simultaneously could be an option to reduce outgrowth of antigen escape variants. One of the approaches to achieve dual targeting is the use of tandem CARs. Different tandem CARs have already been reported [8,23,24,25]. However, the generation of tandem CARs can be challenging due to potential cross pairing between the V_H_ and V_L_ of the different scFvs [26].

Nanobodies can potentially be an alternative antigen-binding moiety. Nanobodies, first described by Hamers-Castermans, are isolated from the V_H_ domain of heavy-chain only antibodies (HcAbs) found in Camelidae [27]. The structure of these antibodies comprises two heavy-chains in which the constant heavy 1 (CH1) domain is lacking. Due to the absence of this domain, they are devoid of a light chain. As a result, the specificity of these HcAbs relies only on one variable domain and not two, allowing elegant screening procedures based on direct isolation and sequencing of the DNA encoding the variable heavy homodimers (VHH). This variable domain can be cloned, easily expressed and retains the affinity for its specific antigen, which is comparable to traditional scFvs. Furthermore, nanobodies are only weakly immunogenic due to their high degree of sequence identity with the human V_H_ gene family III and their small size [28,29]. No or low immune response against a nanobody was raised in mice or humans that were injected with nanobody containing constructs [30,31,32,33]. In addition, Vincke et al. have developed a humanized scaffold nanobody onto which the antigen-binding complementary determining region (CDR) loops can be grafted [34]. Finally, nanobodies are strictly monomeric structures and will not interact with one another [29,35]. Therefore, incorporating nanobodies in the CAR structure could result in a lower probability of CAR clustering on the cell membrane. Furthermore, due to the strict monomeric behavior they are ideal building blocks for multidomain constructs [36]. Both single and tandem nanobody based CARs have been generated earlier and shown similar functionality as scFv-based CARs [37,38,39,40,41,42]. Moreover, a recent clinical trial using CAR T cells expressing a nanobody-based tandem CAR targeting two different epitopes of the B cell maturation antigen (BCMA) showed a remarkable overall response rate of 88.2% in relapsed/refractory myeloma patients [43].

We previously described the use of a dual specific CAR based on nanobodies [42]. To expand the usage of nanobodies in the generation of CAR T cells, here, we report an optimized protocol to speed up the process of generating CAR constructs. 

## 2. Results

### 2.1. CAR Construction

**Figure 1:**
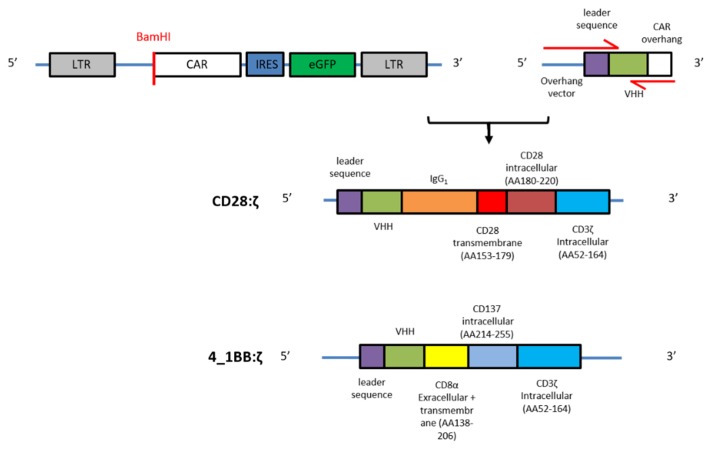
Generation of the nanoCAR constructs: schematic representation of the retroviral CAR backbone plasmid, nanobody with leader sequence and overhangs at 5′ and 3′, and the two different nanoCAR constructs. Red arrows indicate forward and reverse primers.

We designed two retroviral vectors, with each encoding a different second-generation CAR backbone, and one incorporating a CD28 and one a 4_1BB costimulatory domain. The CD28:ζ construct consists of the human IgG_1_ CH2CH3 (Fc) spacer, the CD28 transmembrane domain and the CD28 and CD3ζ intracellular signaling domains. The 4_1BB:ζ construct contains the CD8α hinge and transmembrane region followed by the 4_1BB and CD3ζ intracellular signaling moieties. Both CAR backbone constructs were cloned in a retroviral plasmid that contains an IRES-eGFP sequence to allow easy detection of CAR-expressing cells. At the 5′ position we incorporated a BamHI restriction site (Figure 1). 

We used a CD33 and CD20 nanobody library generated as previously described (see material and methods). The nanobody specific sequences were amplified by PCR using primers with 15–20 nucleotide overhangs complementary to the 5′ retroviral plasmid (forward primer) or the 3′ CAR sequence (reverse primer). The forward primer also encoded a leader sequence derived from the L-kappa murine leader sequence. The PCR products were purified, and Gibson Assembly was used to introduce the amplified sequence into the CAR vector (Figure 1). 

### 2.2. NanoCARs Targeting CD33 are Functional 

The nanobody sequences specific for human CD33 were derived from a llama nanobody phage library. This library was constructed from peripheral blood lymphocytes obtained after immunization of a llama with the extracellular domain of human CD33 protein. Three different nanobody clones were randomly selected and amplified by PCR, purified and cloned into the CAR backbone by Gibson Assembly, resulting in three CD33-specific nanoCAR constructs. After sequence confirmation, we produced retroviral particles and subsequently transduced peripheral blood mononuclear cells (PBMC). NanoCAR expression was confirmed by flow cytometry using an antibody specific for the nanobody protein (Supplementary Figure S1). Transduced T cells were either used as such or sorted for CAR expression and expanded. The frequency of nanoCAR-expressing T cells ranged from 25% to 68% and was equalized before functional testing. 

**Figure 2:**
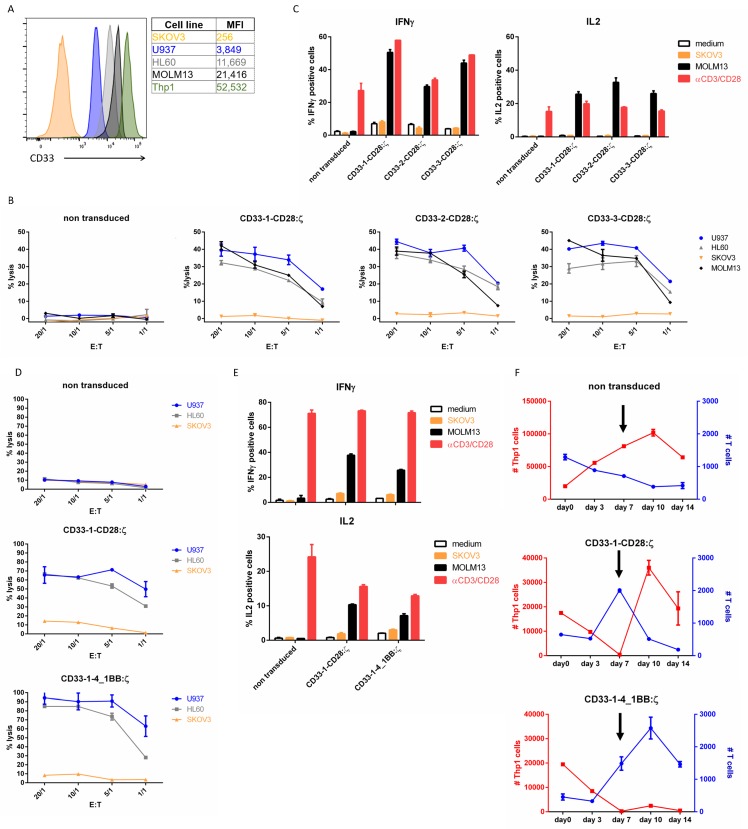
CD33-specific nanoCARs are functional: (**A**) CD33 surface expression on different target cell lines analyzed by flow cytometry. MFI represents median fluorescence index; (**B**) Cell lysis of CD33^+^ or CD33^−^ target cells after 4 h of co-incubation with T cells expressing the CD33-CD28:ζ nanoCAR in different effector-target ratios (E:T). Reported values are the means of duplicate determinations with error bars indicating the standard error of the mean (SEM); (**C**) Cytokine production of nanoCAR T cells was analyzed by intracellular staining 5 h after co-incubation with CD33^+^ or CD33^−^ target cells. Mean percentages of IFN-γ and IL-2 positive cells are shown, gated on eGFP^+^ cells. Error bars indicate the SEM; (**D**) Cell lysis of CD33^+^ or CD33^−^ target cells after co-incubation with CD33-1-CD28:ζ or CD33-1-4_1BB:ζ nanoCAR T cells in different effector-target ratios. Reported values are the means of duplicate determinations. Error bars indicate the SEM; (**E**) Cytokine production of CD33-1-CD28:ζ or CD33-1-4_1BB:ζ nanoCAR T cells. Mean percentages of IFN-γ and IL-2 positive cells are shown. Error bars indicate the SEM; (**F**) Long-term in vitro stress test. NanoCAR T cells (blue) are incubated at very low (0.025:1) effector to target cell (red) ratios. Arrow indicates addition of fresh Thp1 cells at day 7. Error bars represent the SEM. The data are representative of two independent experiments with three different donors. Each experiment shown was performed two times with three different donors. Data shown are representative for these experiments.

Most acute myeloid leukemia cells express CD33. A panel of AML cell lines was analyzed for CD33 expression (Figure 2A). Flow cytometry results confirmed CD33 expression on four AML cell lines (U937, HL60, MOLM13 and Thp1). The CD33^−^ ovarian cancer cell line SKOV3 was used as negative control. 

We tested the activity of all three different nanoCAR constructs in a standard 4 h chromium-51 release assay (Figure 2B). We observed a very potent cytotoxic effect on the three CD33^+^ cell lines. The cytotoxicity was driven by CAR binding to cognate antigens, since CD33^+^ cell lines were neglected by non-transduced T cells and CD33^−^ SKOV3 cells did not elicit target cell lysis. Next, we evaluated cytokine production of our CD33-specific nanoCAR T cells 5 h after co-incubation with our target cell panel by intracellular staining for interferon-γ (IFN-γ) and interleukin-2 (IL-2). The nanoCAR-expressing cells were able to produce both IFN-γ and IL-2 after incubation with CD33^+^ MOLM13 cells. Incubation with the CD33^−^ cell line SKOV3 resulted in no significant expression of IL-2 or IFN-γ (Figure 2C). Altogether, we observed no differences in nanoCAR functionality between the three different nanoCARs both in cytotoxicity and in cytokine profile. 

Next, we compared CD33-specific nanoCARs with different costimulatory domains and spacer lengths. The CD33-1-4_1BB:ζ nanoCAR was highly expressed (Figure S1). Incubation of the nanoCAR T cells with CD33^+^ cells resulted in a potent cytotoxic effect and robust cytokine production (Figure 2D,E). We could not observe differences in short term functionality between the CD33-1-4_1BB:ζ and CD33-1-CD28:ζ CAR T cells. Since 4_1BB signaling in CAR T cells has been described to enhance long-term functionality, we developed a long-term in vitro stress test in which proliferation, survival, cytokine production and serial killing capacity were tested. NanoCAR T cells are incubated at very low effector to target ratios with THP1 cells. After seven days of co-incubation, we re-stimulated the nanoCAR T cells with fresh THP1 cells. At the indicated time points, the number of T cells and target cells present in the co-culture were determined by flow cytometry (Figure 2F). The non-transduced T cells were unable to control the growth of Thp1 cells and dropped in cell numbers at each time point. The CD33-1-CD28:ζ nanoCAR T cells proliferated during the first seven days of co-culture and eliminated all the Thp1 cells present in the co-culture. However, after the addition of fresh Thp1 cells, we observed a strong decline in T cell numbers and the nanoCAR T cells were unable to control the newly added Thp1 cells. T cells expressing the CD33-1-4_1BB:ζ nanoCAR showed similar proliferative activity during the first seven days of co-culture as the CD28:ζ nanoCAR T cells. In contrast to CD28:ζ T cells, proliferation continued until day ten of co-culture. Furthermore, 4_1BB:ζ nanoCAR T cells killed all Thp1 cells even after addition of fresh Thp1 cells at day seven of co-culture. While we observed no differences in short term functionality, we observed a better long-term functionality of the CAR incorporating 4_1BB:ζ signaling. 

### 2.3. NanoCAR T Cells Expressing 4_1BB:ζ Outpreform CD28:ζ NanoCAR T Cells in Vivo

**Figure 3:**
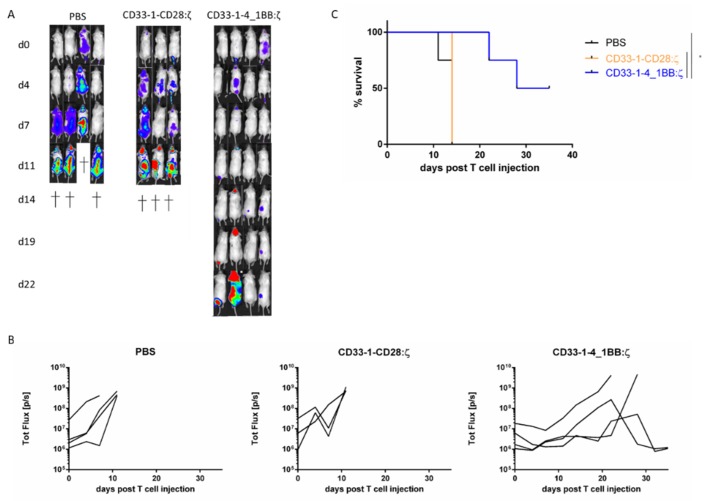
Eradication of CD33^+^ target cells in vivo and improvement of survival by CD33-1-4_1BB:ζ nanoCAR T cells but not by CD33-1-CD28:ζ nanoCAR T cells in a murine xenograft model: (**A**) Bioluminescence images showing tumor burden in NSG mice starting from day 0 post T cell injection; (**B**) Quantification of bioluminescence signal shown in a. Data points shown are the means, and error bars represent the SEM; (**C**) Survival of NSG mice treated with PBS, CD33-1-CD28:ζ or CD33-1-4_1BB:ζ nanoCAR T cells. Only significant results are indicated. * *p* < 0.05 by log-rank Mantel–Cox test.

To evaluate the in vivo potency of CD33-targeted nanoCAR T cell therapy, we used a Thp1 xenograft model. Nonobese diabetic severe combined immunodeficiency gamma (NSG) mice were intravenously injected with Thp1 cells expressing firefly luciferase. The cells were allowed to engraft and expand. After checking engraftment 7 days after injection, a single tail vein injection of nanoCAR T cells was administered (day 0 in Figure 3A). Follow up of the mice was done by serial imaging and disease burden was quantified using bioluminescence. Mice treated with PBS or CD33-1-CD28:ζ nanoCAR quickly succumbed to disease, while mice treated with the CD33-1-4_1BB:ζ showed better survival and a significant reduction in disease burden (Figure 3A–C). We couldn’t find any eGFP^+^ T cells in the spleen, blood or bone marrow of mice injected with CD33-1-CD28:ζ nanoCAR T cells at day fourteen post CAR T cell injection. Two out of four mice treated with CD33-1-4_1BB nanoCAR T cells showed an initial response but relapsed. However, we could not detect Thp1 cells in spleen, bone marrow or blood. This made us believe that these cells were present in anatomical sanctuary sites such as subcutaneous tissue and peritoneum. 

### 2.4. Targeting of CD33 Results in Hematopoietic Toxicity

**Figure 4:**
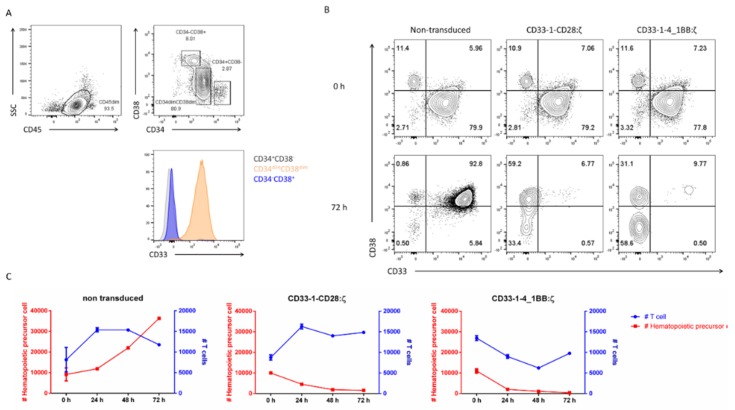
CD33-specific nanoCAR T cells are cytotoxic against CD34^+^ HPC: (**A**) CD33 expression on CD34^+^ HPC isolated from cord blood. CD34^+^ HPC were isolated from cord blood and stained for CD45, CD33, CD34 and CD38. Cells are gated on CD45^dim^SSC^lo^ and CD34^+^CD38^−^, CD34^dim^CD38^dim^ and CD34^−^CD38^+^. Plots are representative for 5 donors; (**B**) Cytotoxicity in time. NanoCAR T cells were incubated with CD34 HPC for 72 h. CD38 and CD33 expression on CD34^+^ HPC measured at the start (zero hour) and the end (72 h) of the experiment; (**C**) Cytotoxicity in time. NanoCAR T cells were incubated with CD34 HPC for 72 h. At distinct time points, we measured the presence of T cells and HPC (gated on CD3^−^) by flow cytometry. Data points shown are the means, and error bars represent the SEM taken from a representative experiment. The experiment was performed two times, each time with two different donors.

CD33 is expressed on myeloid progenitors and CD33-targeted CAR T therapy was reported to cause an on-target off-tumor effect which compromised hematopoiesis [44]. To test whether this was also the case for the nanoCAR T cells, CD34^+^ hematopoietic precursor cells (HPC) were isolated from different cord blood donors and analyzed for CD33 expression. Only CD34^dim^CD38^dim^ HPC expressed CD33 although at a lower level compared with leukemic cell lines (Figure 2A and Figure 4A). CD34^+^ HPC (as shown in Figure 4A) were co-cultured with eGFP transduced or CD33 nanoCAR transduced T cells for 72 h. After 24, 48 and 72 h, we assessed the presence of HPC and T cells by flow cytometry. Non-transduced T cells did not show any toxicity towards the HPC. The HPC started to differentiate from a CD34^+^CD38^−^ towards a CD34^+^CD38^+^ phenotype. This differentiation process was accompanied by a strong proliferation and CD33 upregulation. On the other hand, the CD33 nanoCAR T cells were able to eliminate the majority of the HPC in less than 24 h. A small fraction of the CD34^+^ HPC was still present and had a CD33^−^CD38^+^ phenotype (Figure 4B,C). 

In conclusion, we have shown that it is possible to generate functional CARs using randomly selected nanobodies specific for CD33. We observed a high and stable nanoCAR expression, high cytotoxicity and robust cytokine production when incubated with CD33^+^ cell lines. T cells expressing the 4_1BB:ζ nanoCAR could prolong the survival of NSG mice inoculated with the CD33^+^ Thp1 cell line. As expected, our CD33-specific nanoCARs induced hematopoietic toxicity when co-incubated with CD34^+^ HPC. 

### 2.5. In vitro Evaluation of CD20 NanoCAR T Cells

We next tested our rapid and elegant method of generating nanoCARs for CD20, another clinically relevant antigen. A library was generated from B cells of a llama immunized with DNA encoding for the human CD20 antigen. Three nanobody clones specific for the CD20 antigen were selected and cloned into the 4_1BB:ζ CAR backbone using the method described in 2.1. We used the 4_1BB:ζ CAR backbone only, as it resulted in increased long-term functionality and better in vivo survival of tumor inoculated mice as compared to the CD33-1-CD28:ζ nanoCAR. 

**Figure 5:**
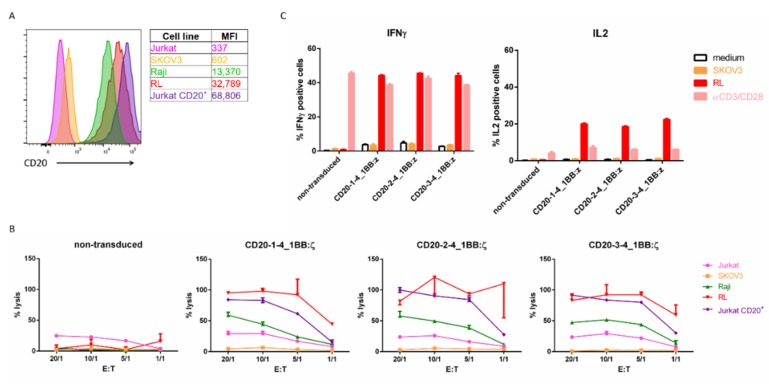
CD20 specific nanoCARs are functional: (**A**) CD20 surface expression on different target cell lines by flow cytometry. MFI represents median fluorescence index; (**B**) Cell lysis of CD20^+^ or CD20^−^ target cells 4 h after co-incubation with T cells expressing CD20 nanoCAR in different effector-target ratios. Reported values are the means of duplicate determinations with error bars indicating the standard error the mean (SEM); (**C**) Cytokine production of nanoCAR T cells was analyzed by intracellular staining 5 h after of co-incubation with CD20^+^ or CD20^−^ target cells. Mean percentages of IFN-γ and IL-2 positive cells are shown, gated on eGFP^+^ cells. Error bars indicate the SEM. Each experiment shown was performed two times with three different donors. Data shown are representative for these experiments.

We analyzed different cell lines for CD20 expression. As expected, the ovarian cancer cell line SKOV3 and T-ALL cell line Jurkat were CD20 negative while the Burkitt lymphoma cell line Raji and non-Hodgkin B lymphoblast cell line RL were CD20 positive. We also included a Jurkat cell line that had a stable transgenic CD20 expression (Figure 5A). 

All three CD20 specific nanoCARs were highly expressed (Figure S1). We first evaluated the cytotoxic activity of the three different CD20 nanoCAR constructs in a standard 4 h chromium-51 release assay. We observed no killing activity of the non-transduced control T cells. However, the CD20 specific nanoCAR constructs vigorously lysed CD20^+^ cell lines while CD20^−^ cell lines remained untouched. We detected some background lysis of the Jurkat cell line, but this was comparable to the non-transduced control T cells and probably caused by alloreactivity (Figure 5B). 

Subsequently, we examined the cytokine production capacity of our CD20 nanoCAR T cells by challenging them with the CD20^−^ SKOV3 cell line and the CD20^+^ RL cell line. After five hours of co-incubation, the cells were harvested and stained intracellularly with antibodies specific for IFN-γ and IL-2. The transduced T cells were able to produce IFN-γ and IL-2 (Figure 5C). There were no significant differences between the different nanoCAR constructs, both in cytotoxicity and in cytokine production (Figure 5B,C). 

### 2.6. NanoCARs Targeting CD20 are Functional in Vivo

**Figure 6:**
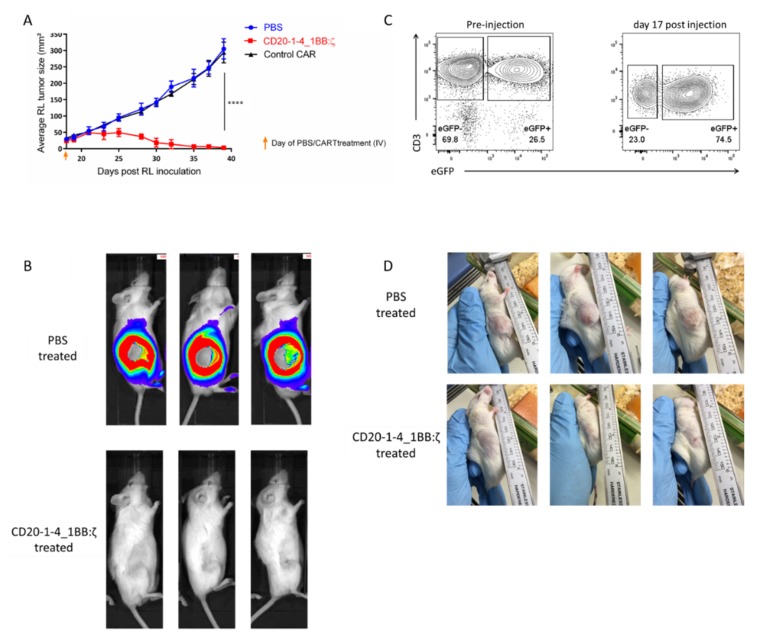
CD20 nanoCAR eradicates CD20^+^ tumor in a murine xenograft model: (**A**) Tumor size measured by caliper. Data points shown are the means, error bars represent the SEM, and only significant results are indicated. **** *p* < 0.0001 by two-way ANOVA with Bonferroni’s multiple comparison test; (**B**) Bioluminescence images showing tumor burden in NSG mice at day 39 post RL inoculation; (**C**) Images showing tumor burden in NSG lice at day 39 post RL inoculation; (**D**) Expression of eGFP in T cells transduced with the CD20-1-4_1BB:ζ naoCAR pre-intravenous injection and in T cells circulating in blood on day 35 post RL injection (day 17 post CAR T cell injection). Plot shown is representative for five mice.

Having determined the in vitro activity of our CD20 nanoCAR T cells, we next sought to determine the in vivo antitumor effect. We established a CD20^+^ xenograft mouse model by subcutaneous inoculation of RL cells. We allowed the cells to form a solid mass and after 18 days we intravenously injected CD20-1 nanoCAR T cells or control nanoCAR T cells intravenously. Tumor growth was measured using caliper and tumor surface area was calculated. As shown in Figure 6A, tumor growth was unhindered by the control nanoCAR T cells. However, the CD20 nanoCAR T cells were able to eliminate the complete subcutaneous tumor in less than 20 days (Figure 6A–C) and prolonged the survival of the mice significantly. Mice treated with PBS or control CAR were sacrificed 39 days post RL inoculation while mice treated with CD20 nanoCAR T cells were kept for six weeks post CAR T injection, until they had to be sacrificed according to ethical guidelines, due to graft versus host disease. During this period, we did not observe any outgrowth of residual tumor cells. The elimination of the RL tumor was driven by nanoCAR-expressing T cells since we observed a significant increase in eGFP^+^ T cells (Figure 6D). In the mice treated with the control nanoCAR T cells, we could not observe any CD3^+^ cells in the blood. 

## 3. Discussion

Here, we describe a protocol for the easy and efficient generation of nanobody based CARs against two different antigens. We generated nanoCARs targeting two clinically relevant antigens, CD20 and CD33, starting from standard second-generation CAR structures and a library of nanobodies targeting the selected antigens. We were able to clone six out of six randomly selected nanobody clones into different second-generation CARs by one single PCR and Gibson Assembly reaction. The nanoCARs were highly expressed and nanoCAR-expressing T cells were functional in vitro. Furthermore, nanoCAR T cells were able to eradicate established CD20^+^ tumors and engrafted CD33^+^ leukemic cells in NSG mice. 

Although these nanoCARs show efficacy both in vivo and in vitro, some optimization and modification are needed to translate these nanoCARs into the clinic. Firstly, although nanobodies are demonstrated to be non-immunogenic in humans, humanization should still be performed. Vincke et al. have proposed a humanized VHH scaffold in which the antigen-binding loops can be grafted [34]. Next, while both lenti- and γ-retroviral vectors are used in clinical trials, there is a preference for lentiviral vectors due to their better safety profile [12,45,46]. We used γ-retroviral particles to transduce stimulated PBMC as it is still considered safe for the transduction of T cells. If we want to translate our nanoCAR T cells into the clinic, we will clone our nanoCAR in a lentiviral vector. Lastly, we used an IRES-eGFP sequence to easily detect our nanoCAR T cells. This sequence should be deleted before use in humans since eGFP can be immunogenic [47]. This immunogenicity could result in killing of our nanoCAR T cells and concomitant loss of nanoCAR T cell efficacy. Our nanoCAR T cells could easily be detected by flow cytometry after staining with a VHH specific antibody. 

To our knowledge, it is the first time that nanobodies are cloned in a CAR backbone using Gibson Assembly. Standard cloning protocols for nanoCARs consist of PCR amplification of the nanobody sequence, sub cloning in a multi-purpose cloning vector, sequencing and eventually cloning into the retro- or lentiviral vector containing the CAR backbone. In our hands, this process could take up several weeks. Our technique using PCR and Gibson Assembly, allowed us to clone several nanobodies in less than four days. 

For the CD33 antigen, we tested two different CAR backbones in vitro. Both CD33-specific nanoCARs induced similar T cell activation, cytokine production and tumor cell lysis when incubated with CD33^+^ cells. On the other hand, in long-term in vitro stress test assays, the 4_1BB:ζ nanoCAR transgenic T cells outperformed the IgG_1_/CD28:ζ nanoCAR transgenic T cells. This is in line with reports from clinical trials. Several studies have shown that the use of a CD28 or a 4_1BB co-stimulatory domain most consistently affects CAR T cell behavior. CAR T cells expressing a CD28 intracellular domain undergo a more rapid and higher peak expansion, but rarely persist for longer than two months. On the other hand, CARs incorporating 4_1BB signaling show a slower and lower peak expansion, but persist for months or even years [2,3,7,9,13]. The underlying biological processes are still unclear, but a quicker and stronger phosphorylation pattern of CD28:ζ CARs after activation has been reported [48]. Next to the signal strength differences, also changes in mitochondrial biogenesis and cell metabolism have been described [49]. 

Besides the differences in intracellular signaling domains, there is also a difference in extracellular spacer region: IgG_1_ in the CD28:ζ nanoCAR versus CD8α hinge in the 4_1BB:ζ nanoCAR. The first being categorized as a long spacer, whereas CD8α, which contains three times less amino acids than the IgG1 spacer, is labelled as a short spacer. Several studies have demonstrated that the length of the spacer is crucial for the formation of the immunological synapse and for the activity of the nanoCAR T cell. When a CAR interacts with its cognate antigen, the CAR T cell has to be at an optimal distance from the target cell. This optimal distance is determined by the length of the spacer domain and the epitope location targeted by the antigen-binding domain. Long spacers provide the most favorable targeting to membrane-proximal epitopes, while CARs bearing a short hinge region are more effective at targeting membrane-distal epitopes [50,51,52,53]. However, we believe that not the spacer but rather the intracellular signaling domain influences the efficacy of our nanoCAR T cells, since in short term experiments we did not observe great differences in activity but the 4_1BB:ζ nanoCAR T cells outperformed the CD28:ζ nanoCAR T cells in our long-term in vitro stress test. 

We also noticed no survival benefit nor disease control in NSG mice injected with CD33-1-CD28:ζ nanoCAR T cells. Furthermore, we were not able to detect any eGFP^+^ T cells in these mice at fourteen days after injection. Different groups have reported on the capacity of human IgG to bind both murine and human Fcγ receptors (FcγR) through their CH2 domain. Earlier studies of the IgG_1_ Fc spacer showed off-target CAR T cell activation by FcRγ^+^ myeloid and lymphoid cells and speculated that potential activation-induced cell death (AICD) could occur [54]. Other groups have shown that the interaction between the IgG spacer and FcγR expressing murine myeloid cells, present in the lung, sequestered the CAR T cells in the lung and activated the CAR T cells through interactions with the Fc portion of the CAR. This resulted in AICD and loss of anti-tumor function [53,55,56,57]. It is therefore possible that our CD33-1-CD28:ζ nanoCAR T cells were captured in the lung and underwent AICD. As a result, they were not detectable in blood, spleen and bone marrow and were unable to execute their anti-leukemia effects. 

In summary, we have shown that our technique of using nanobodies, PCR and Gibson Assembly is a rapid and efficient way to generate nanoCAR T cells with a 100% success rate for the six randomly selected nanobody clones. We chose two completely different antigens: CD20, a tetraspanner and CD33, a single pass receptor, to test our technique. We strongly believe that the use of nanobodies is advantageous over the use of scFvs, since nanobodies are monomeric structures that (i) will probably not aggregate on the T cell surface and therefore not induce premature T cell activation and exhaustion [19]; (ii) will not lose affinity, a possible and known side effect in the design of scFvs [14,15]. 

## 4. Materials and Methods 

### 4.1. Generation of the CD33 and CD20 Library

Procedures for immunization of llamas, preparation of mRNA, construction of the library, and panning were performed as previously described [58].

### 4.2. Culture of Cell Lines

All the cell lines were cultured as per American Type Culture Collection (ATCC, Manassas, Virgina) recommendations. RL, Raji, MOLM13, U937, were cultured in RPMI (Gibco, Invitrogen, Merelbeke, Belgium), supplemented with 10% fetal calf serum (FSC, Gibco, Invitrogen), 2 mM L-glutamine (Gibco, Invitrogen), 100 IU/mL penicillin (Gibco, invitrogen) and 100 IU/mL streptomycin (Gibco, Invitrogen). Jurkat and HL60 were cultured in IMDM (Gibco, Invitrogen) supplemented with 10 % fetal calf serum (FSC, Gibco, Invitrogen), 2 mM l-glutamine (Gibco, Invitrogen), 100 IU/mL penicillin (Gibco, invitrogen) and 100 IU/mL streptomycin (Gibco, Invitrogen) (complete IMDM, cIMDM). SKOV3 was cultured in DMEM (Gibco, Invitrogen) supplemented with 10% fetal calf serum (FSC, Gibco, Invitrogen), 2 mM L-glutamine (Gibco, Invitrogen), 100 IU/mL penicillin (Gibco, invitrogen) and 100 IU/mL streptomycin (Gibco, Invitrogen). Thp1 was cultured in RPMI (Gibco, Invitrogen) supplemented with 0.05 mM β-mercaptoethanol, 10% fetal calf serum (FSC, Gibco, Invitrogen), 2mM L-glutamine (Gibco, Invitrogen), 100 IU/mL penicillin (Gibco, invitrogen) and 100 IU/mL streptomycin (Gibco, Invitrogen).

### 4.3. Generation of NanoCAR Plasmids

The different constructs, as shown in Figure 1, were generated by Gibson Assembly. The two CAR backbone constructs were ordered as gBlock (IDT, Leuven, Belgium) and cloned into the LZRS-IRES-eGFP retroviral plasmid by Gibson Assembly (NEBuilder HiFi DNA Assembly Master Mix, NEB, Ipswitch, MA, USA). The nanobody specific sequences were amplified using PCR. PCR products were visualized on gel and purified using the MinElute PCR Purification kit (Qiagen, Venlo, The Netherlands) as per fabricator instructions. The LZRS-CAR-IRES-eGFP plasmids were overnight digested with BamHI (NEB) and purified using the Zymoclean Large Fragment DNA Recovery Kit (Zymo, Irvine, CA, USA) according to manufacture instructions. Subsequently, digested and purified plasmid was dephosphorylated (rAPid Alkaline Phosphatase, Roche, Vilvoorde, Belgium) and used in a Gibson Assembly reaction together with the purified PCR products. The Gibson Assembly reaction mix was transformed in bacteria (NEB Stable Competent *Escherichia coli* (High Efficiency), NEB) and plated on agar. After overnight incubation, colonies were selected and grown in liquid lysogenic broth (BD Difco, Erebodegem, Belgium) overnight. Plasmids were isolated and sequenced. Colonies containing the correct plasmid were further cultured and midipreps (Qiagen) were performed. 

### 4.4. Generation of Retroviral Particles

Viral particles were produced using the Phoenix A packaging cell line. Phoenix A cells were seeded at 7.5 × 10^5^ cells per 6 cm dish (BD Falcon, Erebodegem, Belgium) and placed overnight in a 7% CO_2_ incubator at 37 °C. Next day, the plasmids encoding the nanoCAR constructs were transfected using calcium phosphate as follows: per 6 cm dish, 10 µg plasmid DNA was diluted in 36 µL 2M CaCl_2_ (homemade) and subsequently nuclease free water (Ambion, Merelbeke, Belgium) was added to a total volume of 300 µL. The DNA-CaCl_2_ solution was pipetted dropwise into a 15 mL polystyrene tube containing 300 µL 2× HEPES-buffered saline (HBS) solution (homemade) while blowing air bubbles in the 2× HBS buffer. The mixture was incubated for 15 min at room temperature and then added dropwise to the cells. Ten minutes before the addition of the DNA mixture, 2 mL medium was added to the cells containing 1 µL of a 200 mM chloroquine (Sigma-Aldrich, Diegem, Belgium) solution. Cells were placed in a 7% CO_2_ incubator at 37 °C overnight. Medium was refreshed at day one. At day two, cells were analyzed for transfection efficiency and transgene expression and reseeded in a T75 culture flask (Falcon) in selection medium containing 2 µg/mL puromycin (Sigma). After an additional two days, selection medium was replaced by medium without puromycin. At day six and day 10, the selection cycle was repeated. Finally, at day fourteen, retroviral supernatant was collected and was frozen at −80 °C until use. Phoenix A cells were analyzed for nanoCAR and eGFP expression at days of reseeding and at day fourteen. 

### 4.5. Generation of NanoCAR-expressing Human T Cells

Buffy coats from healthy donors were obtained from the Belgian Red Cross and used following the guidelines of the Medical Ethical Committee of Ghent University Hospital, after informed consent had been obtained, in accordance with the Declaration of Helsinki. PBMC were isolated by Lymphrop (Axis-shield, Dundee, UK) gradient centrifugation. The percentage of CD3^+^ cells was determined by flow cytometry and T cells were stimulated with Immunocult Human CD3/CD28/CD2 T cell activator (StemCell Technologies, Vancouver, Canada) per fabricator instructions in cIMDM, in the presence of 10 ng/mL IL-12 (PeproTech, Hamburg, Germany). Cells were harvested 72 h after stimulation and resuspended in retroviral supernatant and centrifuged for 90 min at 1000× *g* at 32 °C on retronectin (TaKaRa, Saint-Germain-en-Laye, France) coated plates. 

Transduced cells were detected by eGFP expression and after staining with a nanbody specific antibody. Transduced cells were sorted and expanded on irradiated allogenic feeder cells, consisting of a mixture of 40 Gy irradiated peripheral blood mononuclear cells and 50 Gy irradiated JY cells. Cells were cultured in cIMDM, supplemented with 1 μg/mL phytohemagglutinin (PHA, Sigma–Aldrich. IL-2 (40 IU/mL) (Roche) was added on day five and day ten. Cells were restimulated every seven–fourteen days.

### 4.6. Flow Cytometry and Antibodies

Staining of surface markers was performed as described earlier [42]. The following anti-human monoclonal antibodies were used: phycoerythrin (PE)-conjugated CD33 (MACS Milteyni Biotec, Leiden, The Netherlands); PE-Cy7-conjugated CD3 (eBioscience, Vienna, Austria); allophycocyanin (APC)-conjugated CD38 (Biolegend, London, UK); APC-Fire 750-conjugated CD8α (Biolegend), CD20 (BD Biosciences, Erebodegem, Belgium); BV510-conjugated CD45 (BD Biosciences); Peridnin-chlorophyll Cy 5.5-conjugated CD4 (Biolegend); iFluor 555-conjugated MonoRab Rabbit Anti-Camelid VHH (Genscript by Bio-Connect, Huissen, The Netherlands); Vioblue-conjugated CD34 (MACS Milteyni Biotech). 

### 4.7. ^51^Chromium Release Assay

Cytotoxicity assay was performed as previously described [42]. The following target cells were used for the CD33 nanoCARs: SKOV3, U937, HL60, Thp1 and Molm13; for the CD20 nanoCARs: Jurkat, Jurkat CD20^+^, Raji, RL and SKOV3. 

### 4.8. Flowcytometric Determination of Cytokine Production

Detection of cytokine producing cells was performed as previously described [42], with the following modifications: incubation of target cells with nanoCAR T cells lasted for five hours and the staining for TNF-α was omitted. The target cells used for the CD33 nanoCARs were SKOV3 and Molm13 and for the CD20 nanoCARs RL and SKOV3. 

### 4.9. In Vitro Stress Test 

T cells expressing nanoCARs were incubated with Thp1 cells at an effector/target ratio of 0.025:1 (400 T cells: 2 × 10^4^ Thp1 cells) in cIMDM. Cells were stained with CD3, CD4 and CD8α at the start of the co-culture and at day three, seven, ten and fourteen. At day seven of co-culture, 2 × 10^4^ Thp1 cells were added to the remaining wells. Cell numbers were determined by flow cytometry. 

### 4.10. Tumor Mouse Model

NSG mice were injected intravenously with 2 × 10^6^ Thp1 firefly luciferase^+^ cells, twelve hours after irradiation with 200 cGray. Twelve days post Thp1 injection, in vivo imaging (IVIS, Perkin Elmer, Zaventem, Belgium) was performed after intraperitoneal injection of 150 mg/kg bodyweight d-luciferin (Perkin Elmer) and 5 × 10^6^ nanoCAR T cells were intravenously injected. Tumor progression was followed up by IVIS imaging. Mice were checked for overall health status and scarified when humane endpoints were reached. 

For the RL model, 2 × 10^6^ RL cells were subcutaneously injected. The cells were allowed to form a solid mass and at day eighteen, 5 × 10^6^ nanoCAR T cells were intravenously injected. Tumor progression was followed up by caliper. Mice were checked for overall health status and scarified when human endpoints were reached. 

### 4.11. In vitro Hematopoietic Cytotoxicity

Cord blood was acquired through the Belgian Red Cross and used following the guidelines of the Medical Ethical Committee of Ghent University Hospital (EC/UZG 2015/0768), after informed consent had been obtained, in accordance with the Declaration of Helsinki. PBMC were isolated using density gradient centrifugation. CD34^+^ HPC were purified using CD34 MicroBead Kit (MACS Miltenyi Biotech) per manufactures instructions. T cells expressing the nanoCARs were cultured with CD34^+^ HPC in cIMDM supplemented with stem cell factor (SCF, PeproTech), thrombopoietin (TPO, PeproTech) and FMS-like tyrosine kinase 3 ligand (Flt3-L, MACS Miltenyi Biotech), all three at 100 ng/mL with PBMC at a 1:1 ratio. At the start and at 24 h, 48 h and at 72 h of the co-culture, cells were harvest and stained for CD3, CD34, CD38 and CD33. Subsequently, cells were analyzed by flow cytometry and absolute cell counts were determined.

### 4.12. Sequences and Primers

Underlined sequences indicate overlap with the LZRS vector, cursive sequences indicate BamHI site, sequences in bold indicate leader sequence, sequences in upper case indicate overlap with nanobody sequences and sequences in upper case and bold indicate overlap with CAR backbone.

Nanobody specific sequence amplification.

Fw primer:

5′gggtggaccatcctctagactgcc*ggatcc*gcc**atggattttcaggtgcagattttcagcttcctgctaatcagtgcctcagtcataatgtctaga**CAGGTGCAGCTGCAGGAG3′

Rev primer 4_1BB:ζ backbone:

5′GGTCGCGGCGCTGGCGTCGTGGTC*ggatcc***ACTGAGGAGACGGTGACCTG**3′

Rev primer CD28:ζ backbone:

5′AGGAGATTTGGGCTCGGCGGGC*ggatcc***ACTGAGGAGACGGTGACCTG**3′

DNA sequence of CD28:ζ CAR backbone construct:

gggtggaccatcctctagactgcc*ggatcc*gcc**atggattttcaggtgcagattttcagcttcctgctaatcagtgcctcagtcataatgtctagaGT*ggatcc*CGCCGAGCCCAAATCTCCTGACAAAACTCACACATGCCCACCGTGCCCAGCACCTGAACTCCTGGGGGGACCGTCAGTCTTCCTCTTCCCCCCAAAACCCAAGGACACCCTCATGATCTCCCGGACCCCTGAGGTCACATGCGTGGTGGTGGACGTGAGCCACGAAGACCCTGAGGTCAAGTTCAACTGGTACGTGGACGGCGTGGAGGTGCATAATGCCAAGACAAAGCCGCGGGAGGAGCAGTACAACAGCACGTACCGGGTGGTCAGCGTCCTCACCGTCCTGCACCAGGACTGGCTGAATGGCAAGGAGTACAAGTGCAAGGTCTCCAACAAAGCCCTCCCAGCCCCCATCGAGAAAACCATCTCCAAAGCCAAAGGGCAGCCCCGAGAACCACAGGTGTACACCCTGCCCCCTTCCCGGGATGAGCTGACCAAGAACCAGGTCAGCCTGACCTGCCTGGTCAAAGGCTTCTATCCCAGCGACATCGCCGTGGAGTGGGAGAGCAATGGGCAGCCGGAGAACAACTACAAGACCACGCCTCCCGTGCTGGACTCCGACGGCTCCTTCTTCCTCTACAGCAAGCTCACCGTGGACAAGAGCAGGTGGCAGCAGGGGAACGTCTTCTCATGCTCCGTGATGCATGAGGCTCTGCACAACCACTACACGCAGAAGAGCCTCTCCCTGTCTCCGGGTAAAAAAGATCCCAAATTTTGGGTGCTGGTGGTGGTTGGTGGAGTCCTGGCTTGCTATAGCTTGCTAGTAACAGTGGCCTTTATTATTTTCTGGGTGAGGAGTAAGAGGAGCAGGCTCCTGCACAGTGACTACATGAACATGACTCCCCGCCGCCCCGGGCCCACCCGCAAGCATTACCAGCCCTATGCCCCACCACGCGACTTCGCAGCCTATCGCTCCCTGAGAGTGAAGTTCAGCAGGAGCGCAGACGCCCCCGCGTACCAGCAGGGCCAGAACCAGCTCTATAACGAGCTCAATCTAGGACGAAGAGAGGAGTACGATGTTTTGGACAAGAGACGTGGCCGGGACCCTGAGATGGGGGGAAAGCCGAGAAGGAAGAACCCTCAGGAAGGCCTGTACAATGAACTGCAGAAAGATAAGATGGCGGAGGCCTACAGTGAGATTGGGATGAAAGGCGAGCGCCGGAGGGGCAAGGGGCACGATGGCCTTTACCAGGGTCTCAGTACAGCCACCAAGGACACCTACGACGCCCTTCACATGCAGGCCCTGCCCCCTCGATAA**

DNA sequence of 4_1BB:ζ CAR backbone construct:

gggtggaccatcctctagactgcc*ggatcc*gcc**atggattttcaggtgcagattttcagcttcctgctaatcagtgcctcagtcataatgtctagaGT*ggatcc*GACCACGACGCCAGCGCCGCGACCACCAACACCGGCGCCCACCATCGCGTCGCAGCCCCTGTCCCTGCGCCCAGAGGCGTGCCGGCCAGCGGCGGGGGGCGCAGTGCACACGAGGGGGCTGGACTTCGCCTGTGATATCTACATCTGGGCGCCCTTGGCCGGGACTTGTGGGGTCCTTCTCCTGTCACTGGTTATCACCCTTTACTGCAAACGGGGCAGAAAGAAACTCCTGTATATATTCAAACAACCATTTATGAGACCAGTACAAACTACTCAAGAGGAAGATGGCTGTAGCTGCCGATTTCCAGAAGAAGAAGAAGGAGGATGTGAACTGAGAGTGAAGTTCAGCAGGAGCGCAGACGCCCCCGCGTACCAGCAGGGCCAGAACCAGCTCTATAACGAGCTCAATCTAGGACGAAGAGAGGAGTACGATGTTTTGGACAAGAGACGTGGCCGGGACCCTGAGATGGGGGGAAAGCCGAGAAGGAAGAACCCTCAGGAAGGCCTGTACAATGAACTGCAGAAAGATAAGATGGCGGAGGCCTACAGTGAGATTGGGATGAAAGGCGAGCGCCGGAGGGGCAAGGGGCACGATGGCCTTTACCAGGGTCTCAGTACAGCCACCAAGGACACCTACGACGCCCTTCACATGCAGGCCCTGCCCCCTCGCTAA**

### 4.13. Data Analysis and Statistics

Data were analyzed with GraphPad Prism Software (San Diego, CA, USA). Statistical differences between groups or conditions were determined by two-way ANOVA, followed by Bonferroni post hoc test. Survival curves were compared using the log-rank Mantel–Cox test.

## Supplementary Materials

Supplementary materials can be found at https://www.mdpi.com/1422-0067/21/3/883/s1.

## Abbreviations

AML	Acute myeloid leukemia
APC	Allophycocyanin
BCMA	B cell maturation antigen
CAR	Chimeric antigen receptor
CD	Cluster of Differentiation
CH	Constant Heavy
eGFP	enhanced Green Fluorescent Protein
EMA	European Medicines Agency
FDA	Federal and Drug Administration
Flt3L	FMS-like tyrosine kinase 3 ligand
HBSHcAbs	HEPES-buffered salineHeavy-chain antibodies
HPC	Hematopoietic precursor cell
VHH	Variable domain of the heavy-chain of HcAbs
IFN-γ	Interferon gamma
IgG	Immunoglobulin G
IL-2	Interleukin 2
IRES	Internal Ribosomal Entry Site
NSG	Nonobese diabetic scid gamma
PBMC	Peripheral blood mononuclear cells
PBS	Phosphate buffered saline
PCR	Polymerase chain reaction
PE	Phycoerythrin
PerCpCy5.5	Peridnin-chlorophyll Cy 5.5
PHA	Phytohemagglutinin
SCF	Stem cell factor
scFv	Single-chain variable fragment
SEM	Standard error of the mean
TPO	Thrombopoietin
V_H_	Variable heavy
V_L_	Variable light
VHH	Variable heavy homodimers
