# Supplementary material for: Rapid and Effective Generation of Nanobody Based CARs using PCR and Gibson Assembly
Source: Int J Mol Sci. 2020 Jan 30;21(3):883. doi: 10.3390/ijms21030883 (PMC7037261; doi:10.3390/ijms21030883)
Supplement: Supplementary file 1 [file ijms-21-00883-s001.pdf]

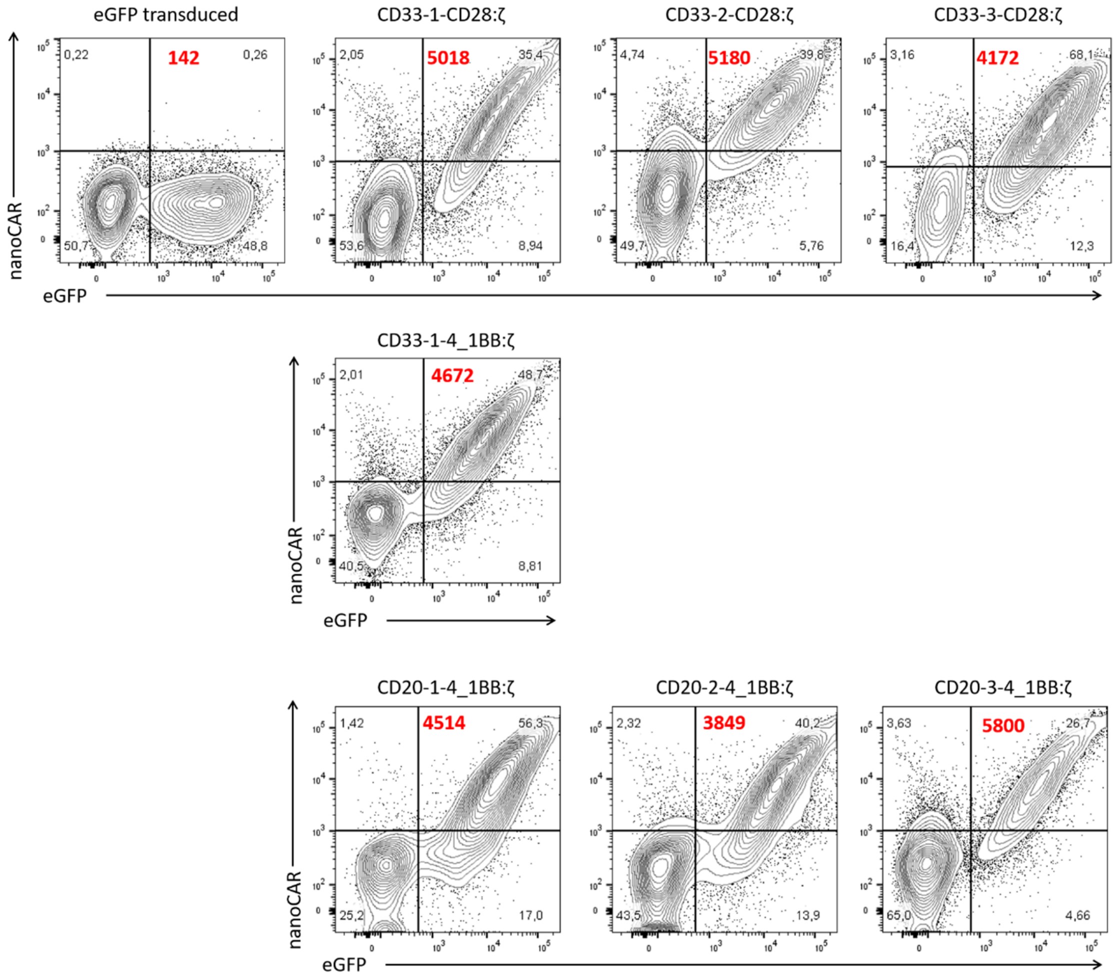

Figure 1. NanoCAR expression was confirmed by flow cytometry using an antibody specific for the nanobody protein. The CD33-1-4_1BB:ζ nanoCAR was highly expressed. All three CD20 specific nanoCARs were highly expressed.
